# Incidence and predictors of continued ascending aortic dilatation after TAVI in patients with bicuspid aortic stenosis

**DOI:** 10.1007/s00392-024-02545-9

**Published:** 2024-09-19

**Authors:** Yuheng Jia, Arif A. Khokhar, Thomas Pilgrim, Giuliano Costa, Darren Mylotte, Sofia Sammartino, Daijiro Tomii, Emil Fosbøl, Corrado Tamburino, Klaus Fuglsang Kofoed, Marco Barbanti, Stephan Windecker, Mao Chen, Ole De Backer

**Affiliations:** 1 The Heart Center, Rigshospitalet, Copenhagen, Denmark; 2 Department of Cardiology, West China Hospital, Chengdu, China; 3 Department of Cardiology, Hammersmith Hospital, Imperial College Healthcare NHS Trust, London, UK; 4 Department of Cardiology and, Cardiovascular Center, Bern University Hospital, Inselspital, University of Bern, Bern, Switzerland; 5 AOU Policlinico ‘G. Rodolico-San Marco’, University of Catania, Catania, Italy; 6 Department of Cardiology, Galway University Hospital, Galway, Ireland; 7 Università Degli Studi Di Enna “Kore”, Enna, Italy

**Keywords:** Aortic stenosis, Bicuspid phenotype, Aortopathy, Aortic dilatation, TAVI

## Abstract

**Background:**

Patients undergoing transcatheter aortic valve implantation (TAVI) for bicuspid aortic stenosis (AS) frequently present with ascending aortic (AAo) dilatation which is left untreated. The objective of this study was to study the natural progression and underlying mechanisms of AAo dilatation after TAVI for bicuspid AS.

**Methods:**

Patients with a native bicuspid AS and a baseline AAo maximum diameter > 40 mm treated by TAVI and in whom post-TAVI computed tomography (CT) scans beyond 1 year were available were included. AAo dilatation was deemed to be either continuous (≥ 2 mm increase) or stable (< 2 mm increase or decrease). Uni- and multivariate logistic regression analysis was utilized in order to identify factors associated with continuous AAo dilatation post-TAVI.

**Results:**

A total of 61 patients with a mean AAo maximum diameter of 45.6 ± 3.9 mm at baseline were evaluated. At a median follow-up of 2.9 years, AAo dimensions remained stable in 85% of patients. Continuous AAo dilatation was observed in 15% of patients at a rate of 1.4 mm/year. Factors associated with continuous AAo dilatation were raphe length/annulus mean diameter ratio (OR 4.09, 95% CI [1.40–16.7], *p* = 0.022), TAV eccentricity at the leaflet outflow level (OR 2.11, 95%CI [1.12–4.53], *p* = 0.031) and maximum transprosthetic gradient (OR 1.30, 95%CI [0.99–1.73], *p* = 0.058).

**Conclusions:**

Ascending aortic dilatation in patients undergoing TAVI for bicuspid AS remains stable in the majority of patients. Factors influencing TAV stent frame geometry and function were identified to be associated with continuous AAo dilatation after TAVI; this should be confirmed in future larger cohort studies.

**Graphical Abstract:**

**Figure:**
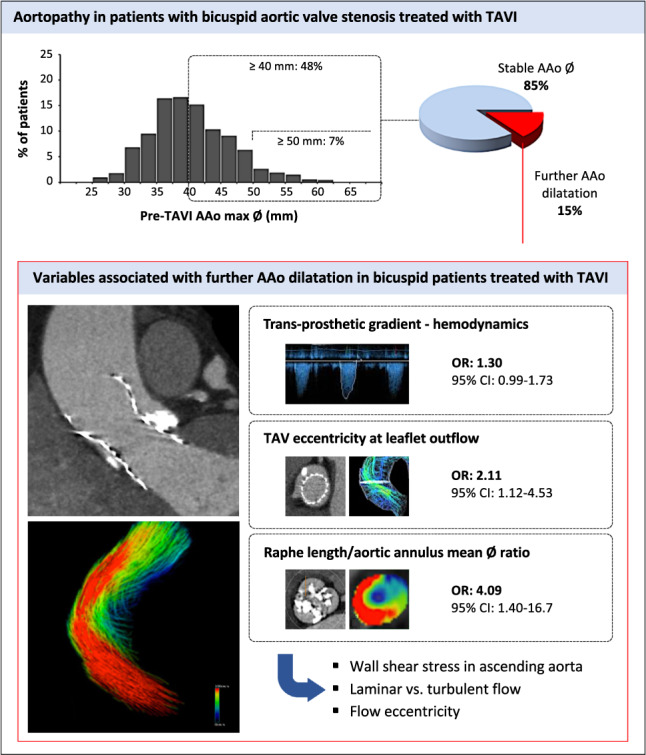
Progression of ascending aortic dilatation in bicuspid aortic stenosis patients who underwent TAVI. Among patients who underwent TAVI for bicuspid aortic valve stenosis, a high prevalence of ascending aortic (AAo) dilatation was observed, which remained stable in 85% of patients at follow-up. In 15% of patients, continuous AAo dilatation was noted and this was associated with an elevated trans-prosthetic maximum gradient, TAV eccentricity at the leaflet outflow level and an elevated ratio of the raphe length/aortic annulus mean diameter. AAo, ascending aorta dilatation; AS, aortic stenosis; CI, confidence interval; CT, computed tomography; max, maximum; OR, odds ratio; TAV, transcatheter aortic valve; TAVI, transcatheter aortic valve implantation; Ø, diameter. Some of the CMR images were used with permission from the copyright owner (Secinaro et al. in Eur Heart J Cardiovasc Imaging 00:1–8, 2021).

## Introduction

Bicuspid aortic valve (BAV) disease is a common cardiac pathology frequently associated with an aortopathy [1, 2]. The development of BAV aortopathy can occur independent of valvular alterations and can lead to pronounced ascending aortic (AAo) dilatation, aneurysm formation and subsequent increased risk for acute aortic events [3–6]. Therefore, current guidelines recommend prophylactic aortic surgery in patients with BAV disease and a dilated aorta with or without valvular dysfunction [7–9].

Transcatheter aortic valve implantation (TAVI) can effectively treat severe symptomatic aortic stenosis (AS). However, in patients with severe bicuspid AS and significant aortopathy undergoing TAVI, the dilated aorta is left untreated. As TAVI expands to younger patients, an increased proportion of BAV disease with associated AAo dilatation is expected to be encountered. [10, 11] In these patients with longer life-expectancy, the causes and long-term consequences of leaving behind a dilated AAo demand attention. Unfortunately, data on this topic is scarce. [12–15]

In this study, we used pre- and post-TAVI computed tomography (CT) scans to evaluate the natural progression in AAo dilatation following TAVI in patients presenting with bicuspid aortic valve stenosis and baseline AAo dilatation.

## Methods

### Study population

**Fig. 1:**
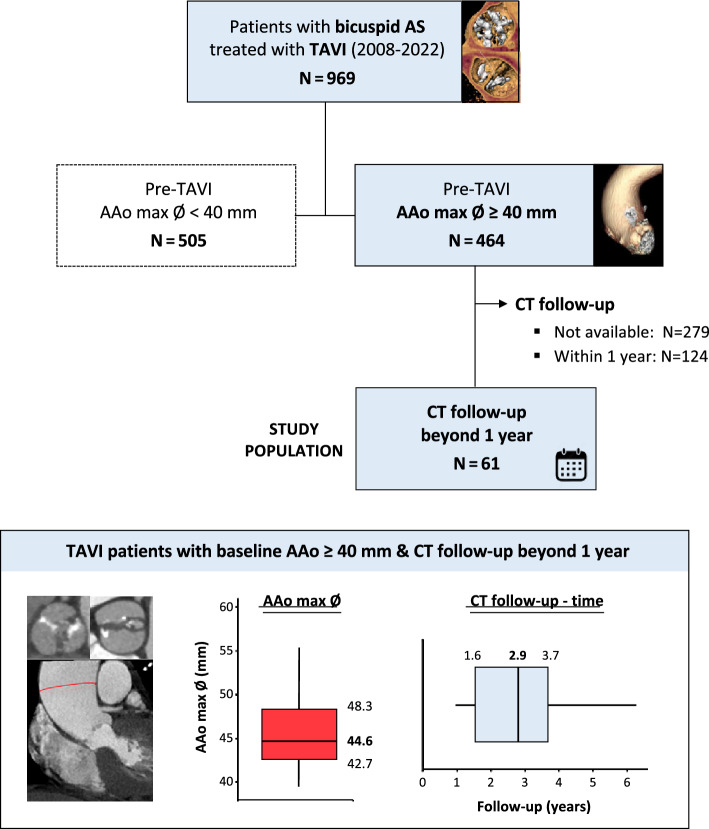
Study population. The study population was derived from a cohort of patients who underwent TAVI for bicuspid aortic stenosis (AS) in whom pre-TAVI computed tomography (CT) identified a baseline ascending aortic diameter > 40 mm. Patients with post-implantation CT beyond 1 year after TAVI were included for analysis. *AAo* ascending aorta; *AS* aortic stenosis; *CT* computed tomography; *max* maximum; *TAVI* transcatheter aortic valve implantation; *Ø* diameter

Among 969 patients who underwent TAVI for bicuspid AS between 2008 and 2022 from four centres (Rigshospitalet, Denmark; AOU Policlinico “G. Rodolico-San Marco”, Italy; Bern University Hospital, Switzerland; West China Hospital, China), pre-TAVI CT was used to identify 464 patients with a dilated aorta, defined as a maximum AAo diameter ≥ 40 mm. [16] Of these, a high-quality post-implant cardiac CT obtained beyond one year was available in 61 patients, which were analyzed for this study (Fig. 1). All cardiac CT scans were electrocardiographically gated, contrast enhanced and thin-sliced (≤ 1.0 mm slice thickness). CT analysis was performed using systolic images, or in the case of motion artefact, the best quality diastolic images. Measurements were all performed and independently verified by physicians experienced in pre- and post-TAVI CT analysis, using 3Mensio software (Pie Medical Imaging, the Netherlands). Patients who underwent TAVI for degenerated surgical bioprosthesis or with a previous history of percutaneous or surgical aortic intervention were excluded. Ethical approval for the study was granted by the local ethical committees and written informed consent was obtained from all patients included.

### Measurement of ascending aortic dilatation

Multi-planar reconstructions were created to measure the minimum, maximum and mean cross-sectional diameters of the sinus of Valsalva (SoV), sinotubular junction (STJ) and the AAo (defined as the tubular aorta arising above the STJ to the level of the pulmonary artery bifurcation) in a plane perpendicular to the central axis of the aorta. The location of the maximum diameter in the AAo was noted with reference to the height from the aortic annular plane to allow for comparison on post-implant CT-scans.

**Fig. 2:**
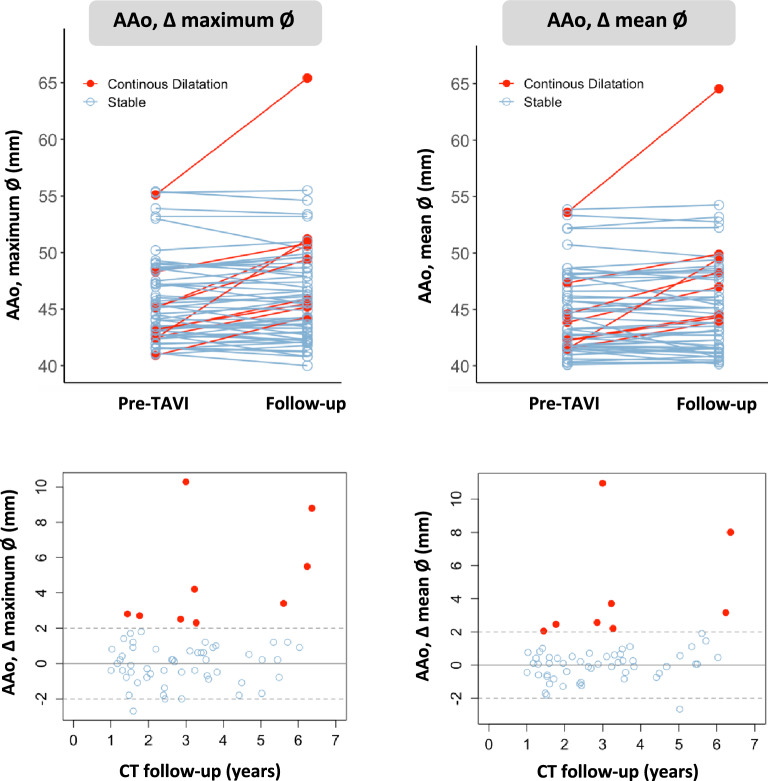
Progression of ascending aortic dilatation after TAVI. Individual patient-level data are presented for the absolute change (Δ) in the maximum and mean AAo diameter after TAVI; this for bicuspid AS patients treated with TAVI and having a baseline maximum AAo diameter > 40 mm. At follow-up, AAo diameters remained stable in the majority of patients. Continuous AAo dilatation after TAVI with a more than 2 mm increase in the maximum and mean AAo diameter was noted in 9 and 8 patients, respectively. *AAo* ascending aorta; *CT* computed tomography; *max* maximum; *TAVI* transcatheter aortic valve implantation; *Ø* diameter

The above measurements were all repeated on the post-TAVI CT-scans. The amount of AAo dilatation post-TAVI was evaluated by calculating the difference in maximum AAo diameter between the pre- and post-TAVI CT. An increase in AAo diameter ≥ 2 mm was considered as continuous dilatation, whereas a decrease or < 2 mm increase in AAo diameter was considered as stable (Fig. 2). The rate of AAo dilatation was calculated as the difference in maximum AAo diameter divided by the time interval in years for each patient.

### Native and transcatheter aortic valve analysis

Pre-TAVI CT scans were used to determine the morphology of the bicuspid aortic valve, measure the raphe length and quantify the volume of calcification (mm^3^) within both the raphe and native aortic valve leaflets [17].

Post-TAVI CT scans were used to evaluate the TAV stent frame geometry. Cross-sectional maximum and minimum TAV stent frame diameters, perimeters and areas were measured at the level of the leaflet inflow and outflow. TAV expansion was calculated as:

$$\text{Expansion}= \frac{\text{measured TAV area}}{\text{nominal TAV area}}$$

TAV eccentricity was calculated using the following formula as previously described [18]:

$$\text{Eccentricity}=\sqrt{1-\frac{{(\text{minimum diameter})}^{2}}{{(\text{maximum diameter})}^{2}}}$$

The value of eccentricity varies between 0 and 1, with a smaller value (closer to 0) representing a more circular shape of the TAV stent frame. Both TAV expansion and eccentricity were measured at the leaflet inflow and outflow levels for each specific TAV.

The TAV performance and hemodynamics were assessed in all patients using transthoracic echocardiography (TTE) prior to discharge from the index hospitalization. The maximum and mean transprosthetic AV gradients and effective orifice area (EOA), calculated using the continuity equation, were obtained.

### Statistical analysis

Categorical variables are expressed as numbers (percentages) and continuous variables as mean ± SD or median (interquartile range). The cohort was divided into two groups: stable AAo and continuous AAo dilatation. Differences between the groups were analyzed using the Chi-square and Student’s t-test, where appropriate. A stepwise uni- and multivariate logistic regression analysis was utilized in order to identify associated factors and independent predictors of continuous AAo dilatation. Clinical, cardiac CT, procedural and post-TAVI echocardiographic variables were included in this analysis. Variables which were associated with continuous AAo dilatation in the univariate model (defined as *p* < 0.1) were included in the multivariate model in order to identify independent predictors of continuous AAo dilatation. For a ratio variable, the odds ratio was expressed for each 0.1 increase in both uni- and multivariate analyses. For maximum transprosthetic AV gradient, the odds ratio was expressed for each 5 mmHg increase. In case of multicollinearity (e.g., raphe length and raphe length/annulus mean diameter ratio), only the variable with the highest statistical power was included in the multivariate analysis. All statistical analyses were performed using SPSS Statistics Software (IBM, NY, USA).

## Results

### Study population

For this study, a total of 61 patients who underwent TAVI for bicuspid AS with a maximum AAo diameter > 40 mm at baseline and who had a cardiac CT at 1–7 years (median 2.9 years) post-procedure could be included. Baseline clinical, CT, procedural and echocardiographic details are summarized in Table 1. The mean age of the study cohort was 73.6 ± 6.6 years, 31.1% were female and the mean surgical risk score was 2.6 ± 1.9%. None of the patients was known with Marfan syndrome or any other connective tissue disorder. Bicuspid aortic valve Sievers type 1 was the most common phenotype in 84% of all patients, with right-left cusp fusion being the predominant BAV type. TAVI was performed using a self-expanding TAV in 67%, a balloon-expandable TAV in 30% and a mechanical TAV in 3% of patients.

**Table 1:** Baseline characteristics

	*N* = 61
Clinical variables	
Age, years	73.6 ± 6.6
Female	19 (31.1%)
Arterial hypertension	47 (77.0%)
Diabetes mellitus	18 (29.5%)
Coronary artery disease	17 (27.9%)
Atrial fibrillation	17 (27.9%)
Chronic renal disease	17 (27.9%)
Peripheral arterial disease	7 (11.5%)
Chronic lung disease	8 (13.1%)
STS risk score, %	2.6 ± 1.9
Baseline CT variables	
Bicuspid aortic valve Sievers type 1	51 (83.6%)
Aortic annulus, mean Ø, mm	26.6 ± 2.9
Sinus of Valsalva, mean Ø, mm	35.5 ± 3.4
Sinotubular junction, mean Ø, mm	34.2 ± 4.1
AAo, mean Ø, mm	44.5 ± 3.8
AAo, maximum Ø, mm	45.6 ± 3.9
TAV prosthesis	
TAV-prosthesis type	
Balloon expandable valve	18 (29.5%)
Self-expanding valve	41 (67.2%)
Mechanical valve	2 (3.3%)
TAV-prosthesis size, mm	28.6 ± 3.8
Post-TAVI echocardiography variables	
Mean AV gradient, mmHg	11.6 ± 6.9
Maximum AV gradient, mmHg	21.9 ± 11.8
Effective orifice area, cm^2^	1.8 ± 0.6
Left ventricular ejection fraction, %	54.3 ± 10.7

*AAo* ascending aorta; *AV* aortic valve; *CT* computed tomography; *STS* Society of Thoracic Surgeons; *TAV* transcatheter aortic valve; *TAVI* transcatheter aortic valve implantation

### Ascending aortic dilatation

At baseline, the maximum AAo diameter was 45.6 ± 3.9 mm and the mean AAo diameter was 44.5 ± 3.8 mm in the overall study cohort. The STJ was also relatively wide with a mean diameter of 34.2 ± 4.1 mm (Table 1).

Over a median study period of 2.9 years (IQR 1.6–3.7 years), the maximum AAo diameter remained stable in 85.2% of patients, whilst continuous AAo dilatation was noted in 14.8% of patients (Fig. 2). A > 5 mm increase in the maximum AAo diameter was observed in three patients and the AAo dilated beyond 55 mm in two patients. Among the nine patients with continuous dilatation based on a maximum AAo Ø expansion ≥ 2 mm, there was calculated an expansion rate of 1.4 mm/year, with the fastest expansion rate being 3.4 mm/year. In contrast the overall expansion rate for the entire cohort was 0.2 mm/year.

### TAV geometry and hemodynamics

At post-implant CT, TAV prostheses were found to be under-expanded and eccentric in shape across the entire cohort. Average TAV expansion at the leaflet inflow and outflow level was 0.65 ± 0.19 and 0.89 ± 0.34, respectively, whilst TAV eccentricity at the leaflet inflow and outflow level was 0.51 ± 0.16 and 0.42 ± 0.12, respectively.

At pre-discharge TTE, the mean and maximum trans-prosthetic valve gradient was 11.6 ± 6.9 mmHg and 21.9 ± 11.8 mmHg, respectively, with a calculated mean EOA of 1.8 ± 0.6 cm^2^ (Table 1).

### Associated factors and predictors of AAo dilatation after TAVI

Multiple clinical, CT, procedural and echocardiographic variables were screened to identify variables associated with AAo dilatation after TAVI. Both statistical analysis using Chi-square and Student’s t test as well as univariate logistic regression analysis could identify the ratio of raphe length/aortic annulus mean Ø, TAV eccentricity at leaflet outflow level and maximum trans-prosthetic gradient as factors associated with continuous AAo dilatation. None of the clinical variables or baseline aortic dimensions were associated factors (Table 2).

**Table 2:** Variables associated with further AAo dilatation in bicuspid patients treated with TAVI

	AAo stable *N* = 52	AAo dilatation *N* = 9	*p* value	Univariate analysis OR (95% CI)	*p*-value
Clinical variables					
Age, years	73.9 ± 6.2	72 ± 8.8	0.441	0.67 (0.24–1.96)	0.436
Female	15 (28.8%)	4 (44.4%)	0.587	1.97 (0.44–8.49)	0.357
Arterial hypertension	41 (78.8%)	6 (66.7%)	0.709	0.54 (0.12–2.86)	0.427
Diabetes mellitus	16 (30.8%)	2 (22.2%)	0.902	0.64 (0.09–3.02)	0.606
Coronary artery disease	15 (28.8%)	2 (22.2%)	0.995	0.70 (0.10–3.33)	0.683
Atrial fibrillation	16 (30.8%)	1 (11.1%)	0.417	0.28 (0.01–1.72)	0.250
Chronic renal disease	15 (28.8%)	2 (22.2%)	0.995	0.70 (0.10–3.33)	0.683
Peripheral arterial disease	7 (13.5%)	0 (0.0%)	0.546	–	–
Chronic lung disease	8 (15.4%)	0 (0.0%)	0.467	–	–
Baseline CT variables					
Aortic annulus, mean Ø, mm	26.5 ± 2.7	27.3 ± 4.1	0.474	1.09 (0.85–1.22)	0.468
Aortic annulus, max/min Ø ratio	1.27 ± 0.10	1.25 ± 0.12	0.571	0.81 (0.37–1.63)	0.565
Sinus of Valsalva, mean Ø, mm	35.6 ± 3.4	35.3 ± 3.3	0.857	0.98 (0.78–1.21)	0.854
Sinotubular junction, mean Ø, mm	34.2 ± 4.4	34.2 ± 2.6	0.996	1.00 (0.85–1.22)	0.996
AAo, mean Ø, mm	44.6 ± 3.8	44.0 ± 4.2	0.670	0.96 (0.77–1.15)	0.664
AAo/ annulus mean Ø ratio	1.70 ± 0.20	1.65 ± 0.30	0.566	0.91 (0.64–1.26)	0.559
AAo, max Ø, mm	45.7 ± 3.8	45.1 ± 4.3	0.674	0.96 (0.77–1.15)	0.669
Bicuspid Type 1	44 (84.6%)	7 (77.8%)	0.981	0.64 (0.12–4.78)	0.611
Raphe length	11.8 ± 2.8	13.9 ± 2.5	0.067	1.34 (0.99–1.92)	0.078
Raphe length/ annulus mean Ø ratio	0.44 ± 0.09	0.54 ± 0.07	0.014	4.09 (1.40–16.7)	0.022*
Raphe calcium, mm^3^	283 ± 312	420 ± 418	0.311	1.12 (0.88–1.38)	0.314
Calcium fused leaflets, mm^3^	616 ± 543	898 ± 625	0.218	1.08 (0.95–1.24)	0.227
TAV prosthesis					
Self-expanding TAV	34 (65.4%)	7 (77.8%)	0.729	1.85 (0.40–13.3)	0.470
TAV prosthesis size	28.6 ± 4.0	28.7 ± 2.4	0.970	1.00 (0.83–1.21)	0.970
TAV post-dilatation	21 (40.4%)	3 (33.3%)	0.976	0.74 (0.14–3.13)	0.690
TAV expansion at leaflet inflow	0.66 ± 0.20	0.63 ± 0.14	0.653	0.91 (0.60–1.32)	0.646
TAV expansion at leaflet outflow	0.89 ± 0.35	0.89 ± 0.35	1.000	1.00 (0.81–1.23)	0.999
TAV eccentricity at leaflet inflow	0.51 ± 0.16	0.53 ± 0.17	0.738	1.08 (0.69–1.76)	0.733
TAV eccentricity at leaflet outflow	0.41 ± 0.12	0.51 ± 0.11	0.022	2.11 (1.12–4.53)	0.031*
Post-TAVI echocardiographic variables					
Mean AV gradient, mmHg	10.9 ± 5.9	15.7 ± 10.4	0.055	1.18 (0.98–1.44)	0.074
Maximum AV gradient, mmHg	20.6 ± 10.4	29.2 ± 17.0	0.044	1.30 (0.99–1.73)	0.058*
Effective orifice area, cm^2^	1.92 ± 0.60	1.53 ± 0.43	0.084	0.52 (0.21–1.05)	0.095
Paravalvular regurgitation ≥ moderate	9 (17.3%)	3 (33.3%)	0.508	2.39 (0.44–11.0)	0.274
Left ventricular ejection fraction, %	53.9 ± 10.9	56.7 ± 10.0	0.486	1.03 (0.96–1.12)	0.479

*AAo* ascending aorta; *AV* aortic valve; *CT* computed tomography; *OR* odds ratio; *TAV* transcatheter aortic valve; *TAVI* transcatheter aortic valve implantation

^*^p value < 0.10 for variable with the highest statistical power in case of multicollinearity

In the multivariate analysis, the ratio of raphe length/aortic annulus mean Ø remained the strongest independent predictor for continuous AAo dilatation (OR 5.67, 95% CI [1.50–35.3], *p* = 0.026); TAV eccentricity (OR 2.20, 95% CI [1.00–6.65], *p* = 0.093) and maximum AV gradient (OR 1.11, 95% CI [0.74–1.64], *p* = 0.598) were less predictive variables (Table 3).

**Table 3:** Independent predictors of further AAo dilatation in bicuspid AS patients treated with TAVR

	Univariate model		Multivariate model	
	OR (95% CI)	*p* value	OR (95% CI)	*p* value
Raphe length/annulus mean Ø ratio	4.09 (1.40–16.7)	0.022	5.67 (1.50–35.3)	0.026*
TAV eccentricity at leaflet outflow	2.11 (1.12–4.53)	0.031	2.20 (1.00–6.65)	0.093
Maximal AV gradient, mmHg	1.30 (0.99–1.73)	0.058	1.11 (0.74–1.64)	0.598

*AAo* ascending aorta; *AS* aortic stenosis; *AV* aortic valve; *CI* confidence interval; *OR* odds ratio; *TAV* transcatheter aortic valve; *TAVR* transcatheter aortic valve replacement

^*^p value < 0.05

## Discussion

In this unique dataset of pre- and post-implant CT scans from patients who underwent TAVI for bicuspid AS with baseline AAo dilatation, the key conclusions are as follows: (1) at a median follow-up of 2.9 years, AAo dimensions remained stable in 85% of patients, (2) continuous AAo dilatation at an average rate of 1.4 mm/year was observed in 15% of patients, and (3) the ratio of the raphe length/mean annulus diameter, TAV eccentricity and maximum AV gradient were associated with continuous AAo dilatation after TAVI (Graphical abstract).

### Ascending aortic dilatation after valve intervention

Historical studies have consistently reported the relative stability in AAo dilatation following surgical aortic valve replacement (SAVR). [19–22] In the largest study of 448 patients with mixed bicuspid and tricuspid AV disease and varying degrees of aortopathy, surgical valve replacement only (sparing the dilated aorta) was associated with no significant increase in AAo dimensions at a mean follow-up of 7.5 ± 3.9 years. [19]

Similarly, prior TTE- and CT-based follow-up studies of patients undergoing TAVI for mixed bicuspid or tricuspid AV disease, reported relative stability in AAo dimensions at medium-term follow-up. [12–15] In a CT-based follow-up study (median follow-up of 1.7 years) of 208 tricuspid and bicuspid AV patients undergoing TAVI with baseline aortic dilatation (mean diameter 41 ± 5.1 mm), a mean increase in AAo diameter of 0.5 ± 1.0 mm and a mean dilatation rate of 0.3 ± 0.8 mm/year was observed. [13] Among 107 patients with moderate-to-severe AAo dilatation (mean diameter 48.6 ± 2.8 mm), the progression rate of AAo dilatation was 0.0 ( – 0.3–0.2) mm/year. [15] Our study complements these findings in a cohort of patients with exclusively bicuspid AS and baseline AAo dilatation. Although stability of AAo dilatation post-TAVI was observed in the majority (85%) of patients, 15% of patients had continuous AAo dilatation (> 2 mm) after TAVI at an average expansion rate of 1.4 mm/year.

### Factors underlying continuous AAo dilatation post-TAVI

Progressive AAo dilatation in patients with bicuspid AV arises due to a complex interaction between the underlying genetics and hemodynamic factors [23–25]. Patients with bicuspid aortic valve disease exhibit abnormal helical and eccentric blood flow patterns, which result in increased aortic wall shear stress (WSS) [26, 27]. The downstream consequences of increased WSS are regional changes in gene expression, dysregulation of the extra-cellular matrix and increased elastic fiber degeneration leading to the characteristic asymmetric AAo dilatation observed in BAV disease [28].

The type, extent and location of these abnormal blood flow patterns can be influenced by the structure and phenotype of the bicuspid valve complex [26, 27] as well as the type of surgical or transcatheter aortic valve intervention [29–31]. Restoration of central laminar blood flow patterns following surgery is associated with improvements in WSS and subsequent stability in AAo dilatation [32–34]. In contrast, persistence of more eccentric helical blood flow patterns and resulting higher WSS values have been reported following surgical implantation of tissue bioprosthetic valve or after TAVI [29–31].

These changes in blood flow patterns following valvular intervention may explain why, in our study, certain variables were associated with continuous AAo dilatation after TAVI. A long and calcified raphe, resulting in an increased raphe/mean annulus diameter ratio, may cause tilting of the assembled TAV complex resulting in a non-central, eccentrically directed jet towards the aortic wall. Similarly, a non-circular eccentric TAV stent frame, at the level of the leaflet outflow, prevents a laminar aortic blood flow, creating abnormal non-laminar blood flow patterns, such as helical flows. These abnormal flow patterns can be further exacerbated by high flow velocities, as measured by higher trans-prosthetic gradients. Consequently, the non-centric, non-laminar and high velocity flow, generated by a sub-optimal structure and function of the implanted TAV, may worsen WSS on the aortic wall leading to continuous AAo dilatation. However, due to the relatively small cohort of patients, these findings should be interpreted as hypothesis-generating and further larger studies are required to determine a pathophysiological link between the assembled structure and function of the implanted TAV, aortic blood flow pattern abnormalities and WSS, and subsequent AAo dilatation.

### Clinical implications

As TAVI expands to younger populations, an increasing proportion of patients with bicuspid AS and AAo dilatation will be encountered [10, 11]. In these patients with longer life-expectancies, the ability to predict, stabilise and monitor AAo dilatation becomes increasingly relevant. In this context, our study sheds new insights. First, baseline CT scans may be used to identify variables predictive of continuous AAo dilatation after TAVI, which may be instructive when trying to determine the optimal lifetime management strategy for younger patients with BAV disease and AAo dilatation [35]. Intra-procedurally, efforts to achieve a well-expanded circular TAV with low residual gradients may be important to ensure optimal aortic blood flow patterns to stabilise AAo dilatation. Finally, in patients with baseline AAo dilatation and unfavourable BAV anatomy or TAV implantation, a close surveillance of the AAo should be considered. However, further studies are warranted to determine the optimal time, duration and imaging modality necessary for monitoring.

### Limitations

Our study is based on a relatively small cohort of patients; therefore, the conclusions should be considered hypothesis-generating and require validation in a larger patient cohort. Due to the small sample size of patients with continuous AAo dilatation, potential other clinical, baseline CT or TAV-related factors associated with continuous AAo dilatation cannot be excluded. Also, systematic genetic testing was not available. The median duration of follow-up was 2.9 years; the longer term AAo diameter stability and natural progression of AAo dilatation up to 5 to 10 years remains unknown. Although no patients with continuous AAo dilatation experienced an adverse aortic event, a selection bias cannot be excluded, as only patients in whom post-TAVI CT scans beyond one year were available were included. Finally, this study only included patients with bicuspid AS; hence, these study findings cannot be translated to patients with bicuspid aortic regurgitation or tricuspid AV disease.

## Conclusions

Ascending aortic dilatation remains stable in the majority of patients undergoing TAVI for bicuspid AS. In 15% of patients, progression in aortic dilatation is observed, which seems to be related to the structure and function of the implanted TAV. The ratio of the raphe length to aortic annulus mean diameter was the strongest predictor for continuous ascending aortic dilatation after TAVI.

## Data Availability

The data that support the findings of this study are available from the corresponding author upon reasonable request.

## Abbreviations

AAo: Ascending aorta
AS: Aortic stenosis
AV: Aortic valve
BAV: Bicuspid aortic valve
CT: Computed tomography
MRI: Magnetic resonance imaging
SAVR: Surgical aortic valve replacement
SoV: Sinus of Valsalva
STJ: Sinotubular junction
TAV: Transcatheter aortic valve
TAVI: Transcatheter aortic valve implantation
WSS: Wall shear stress
